# Potentials and Safety of Date Palm Fruit against Diabetes: A Critical Review

**DOI:** 10.3390/foods9111557

**Published:** 2020-10-28

**Authors:** Md Al-Tareq Mia, Md Golam Mosaib, Md Ibrahim Khalil, Md Asiful Islam, Siew Hua Gan

**Affiliations:** 1 Laboratory of Preventive and Integrative Biomedicine, Department of Biochemistry and Molecular Biology, Jahangirnagar University, Savar, Dhaka 1342, Bangladesh; tareqmia454@gmail.com (M.A.-T.M.); drmikhalil@gmail.com (M.I.K.); 2 Department of Biochemistry and Molecular Biology, Gono University, Savar, Dhaka 1344, Bangladesh; gmosaib@gmail.com; 3 Department of Haematology, School of Medical Sciences, Universiti Sains Malaysia, Kubang Kerian 16150, Kelantan, Malaysia; 4 School of Pharmacy, Monash University Malaysia, Jalan Lagoon Selatan, Bandar Sunway 47500, Selangor, Malaysia

**Keywords:** date palm, *Phoenix dactylifera*, dates, polyphenols, flavonoids, diabetes, anti-diabetic

## Abstract

Diabetes is a chronic metabolic disorder triggered by disturbances in carbohydrate, protein, and lipid metabolisms, where either reduced secretion or sensitivity of insulin is observed coupled with poor glucose control. Date palm fruits are one of the fruits reported to have good potential in diabetes treatment due to its presence of polyphenols exerting strong antioxidant activities. Other possible mechanisms of action include the polyphenolic compounds, which can inhibit enzymes like α-amylase and α-glucosidase. Flavonoids in dates can stimulate β-cells by increasing the number of islets and β-cells, recovering endocrine pancreatic tissues, reducing β-cell apoptosis, activating insulin receptors following the increase in insulin secretion, in addition to improving diabetes-induced complications. In this review, the in vitro, in vivo, and human study-based evidence of date palm as an anti-diabetic fruit is summarised.

## 1. Introduction

Diabetes mellitus (DM) is a disease that is universally emerging, incurring long-standing complexities on organs, including the heart, kidneys, retina, and peripheral nerves. Natural products, which are often free from side effects, are good alternatives for disease amelioration [1,2,3]. Additionally, natural products have potentially effective roles in regulating diabetes and its complications [2,4,5].

Date palm fruit (*Phoenix dactylifera*) is from the Arecaceae or Palmae family. It is one of the oldest cultivated plants and originates from the Arabian Peninsula. There are more than 2000 distinct assortments of dates, including Ajwa, Khalas, Ruthana, Sukkary, Sefri, Segae, Khodry, Lulu, Hilali, and Munifi [6,7]. Dates are very nutritious and are rich in starch, salts, minerals, nutrients, unsaturated fats, proteins and fibres [8,9].

The presence of variable active constituents in dates including flavonoids, steroids, phenol, and saponins are postulated to exert anti-diabetic activities mainly by scavenging the free radicals via antioxidant activities and by inhibiting α-amylase and α-glucosidase enzymes [10,11,12,13]. Fibres and fructose are glucose-lowering, where consumption of dates up to 76.2 g as a snack is purported to be beneficial in Arabic culinary tradition, and can maintain the postprandial glucose levels in patients with diabetes [14,15]. Additionally, the date palm has numerous therapeutic potentials, including cell reinforcement, anti-mutagenic, antibacterial, antifungal, antitumor, neuroprotective, and gastroprotective properties [16,17]. The antioxidant potential in dates is perceived to be contributed by the wide range of phenolic components present, including *p*-coumaric, flavonoids, procyanidins, ferulic, and sinapic acids. Other investigations indicated that the date palm possesses thirteen flavonoid glycosides, including luteolin, quercetin, and apigenin. Ajwa date, also known as a “super date”, and found only in Saudi Arabia, has good nutritional and medicinal values, making it one of the most expensive date varieties [18,19,20].

Although, to date, only a single study systematically reviewed the potentials of date seeds exerting anti-diabetic activity [21], there is no such review to critically assess the potentials and safety of date palm fruit on diabetes. Therefore, the objective of this comprehensive review is to summarise the potential anti-diabetic activities and safety of date palm fruits based on the in vitro, in vivo, as well as human experimental studies.

## 2. Materials and Methods

Different databases, including PubMed, Scopus, and Google Scholar, were searched using the following keywords: *Phoenix dactylifera*, dates, date palm, palm date, date fruit, date fruits, date seed, date seeds, diabetes, diabetic, prediabetic, hyperglycemic, hypoglycemic, hyperglycaemic, and hypoglycaemic. There was no limit in the year of publication and studies published in English were only considered. The final search was conducted on 20 July 2020.

## 3. Physicochemical and Nutritional Composition

Many studies confirmed that date palm fruit is rich in carbohydrates (glucose: 42.3–51.8%, fructose: 22.5–47.5%, sucrose: 3.2–7.4%), proteins (1.8–3.0%), and fibre (2.2%) [22,23]. Date palm seeds were detected with high levels of fatty acids, such as oleic (36.6–50.1%), linoleic (8.9–19.2%), lauric (10.2–20.4%), palmitic (9.8–10.2%), and stearic acids (7.5–10.7%), in addition to some essential oils and major tocols, including α-, β-, and γ-tocotrienols [22,24,25,26]. Minerals, such as potassium, calcium, and magnesium were also detected in date palms [23,24]. Date fruits also contain various types of polyphenols and flavonoids, which are believed to be potential sources of bioactive compounds offering health benefits (Table 1).

## 4. Date Fruit Flesh

### 4.1. Evidence from In Vivo Studies

#### 4.1.1. Antihyperglycemic Activity

**Figure 1:**
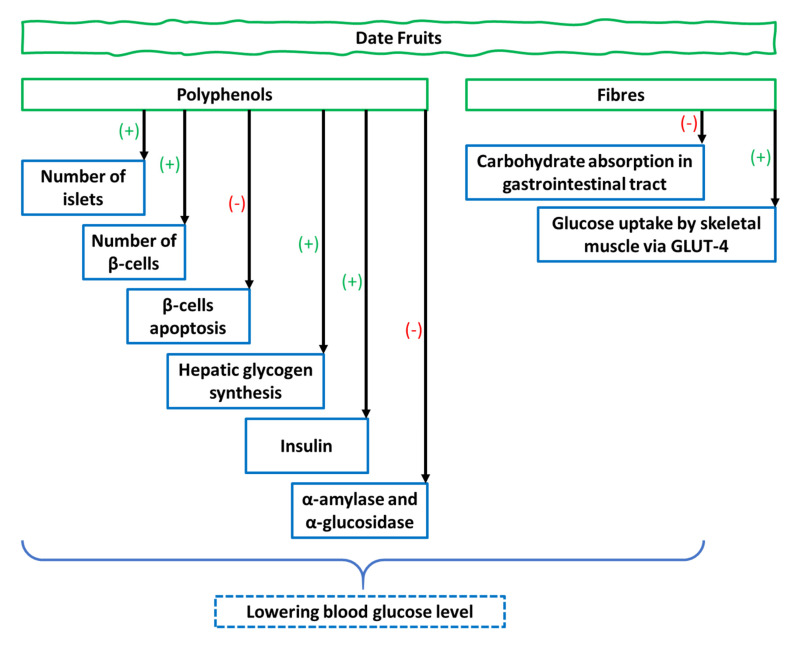
Mechanism of action of polyphenols and fibres of date fruits in ameliorating diabetes.

*Phoenix dactylifera* extracts were administered to streptozotocin-induced type 2 diabetes mellitus (T2DM) rats (*n* = 50) daily for a month, and were found to significantly decrease (*p* < 0.001) glucose levels and increase insulin concentration [46]. *Balanites aegyptiaca* (Heglig dates) reduced blood glucose (209.4 ± 48.2 mg/dL vs. 410.2 ± 45.6 mg/dL) and haemoglobin A1c (HbA1c) levels (8.1 ± 1.4 vs. 6.7 ± 1.5) and improved insulin secretion (1.2 ± 0.3 vs. 1.9 ± 0.3) as compared to the untreated diabetic group [47]. The possible mechanism was through flavonoids, which can increase the number of β-cells and stimulate insulin secretion (Figure 1) [48]. In another 14-day in vivo study [49], *Phoenix dactylifera* fruit pulps were administered to diabetic rats and the dates were found to significantly (*p* < 0.05) reduce blood glucose levels as compared to that for the control and diabetic untreated groups. The possible mechanism is believed to be due to the slowing of gastric emptying by the action of polyphenols found in date fruits (Table 1) [50]. The findings indicate that date fruit pulp has the potential to be used for glucose-lowering. Subsequently, El Abed et al. [10] confirmed that date fruit extract significantly (*p* < 0.01) reduced the plasma glucose level (from 214.0 to 157.2 mg/dL) as compared to acarbose in diabetic albino mice. 

In another study, Aseel or the native variety was orally administered to 32 normoglycaemic and hyperglycaemic Sprague-Dawley rats in two doses (300 and 600 mg/kg). At the end of the experiment, there was insignificant blood glucose reduction in normoglycaemic rats, although these changes were significant (*p* < 0.005) in hyperglycaemic rats (from 281.4 ± 8.0 to 203.2 ± 12.0 mg/dL) [51]. It is plausible that dietary fibres from the date extract reduce carbohydrate absorption in the gastrointestinal tract and affect glucose uptake by skeletal muscle insulin responsive glucose transporter (GLUT)-4 [48]. Additionally, polyphenolic compounds of date extract may have the capacity to inhibit α-amylase and α-glucosidase, which are involved in carbohydrate breakdown, as well as intestinal absorption (Figure 1) [52]. In a 36-day in vivo study on male Sprague–Dawley rats, the animals were divided into three groups (normal, insulin-treated, and insulin-untreated) followed by the oral administration of either 0% or 10% Birhi tamr date variety for six weeks. Ibrahim et al. [53] observed no significant difference in blood glucose levels between normal or insulin-treated rats, although there was significant increase (*p* < 0.05) in insulin-untreated rats; (496.0 ± 81.6 and 315.0 ± 61.1 mg/dL, respectively) compared to normal (147.0 ± 5.3 and 156.0 ± 7.6 mg/dL, respectively) and insulin-treated diabetic rats (227.0 ± 17.6 and 268.0 ± 18.9 mg/dL, respectively) for 0% and 10% Birhi tamr administration. The findings indicate that Birhi tamr date fruit is beneficial for diabetic patients. Interestingly, the authors hypothesized that the antihyperglycemic activity seen was contributed by the presence of insulin-like substance in the Birhi tamr date fruit.

The efficacy of desert dates (*Balanites aegyptiaca*) was evaluated in a 28-day in vivo study, where it was found to significantly (*p* < 0.001) reduce blood glucose levels and body weight in addition to improve insulin secretion, MDA, and liver-pyruvate kinase levels, the size of pancreas, and the islets of Langerhans (*p* < 0.001) [54]. It is plausible that genistein, a flavonoid found in dates reduces β-cells apoptosis, increases β-cells number, promotes β-cells survival in pancreatic islets and preserves islets mass, subsequently increasing insulin secretion while reducing glucose levels due to activated liver-pyruvate kinase [55]. In another six-week in vivo study, Bendary et al. [56] evaluated glucose, insulin and HbA1c levels in albino rats (*n* = 40). At the end of experiment, they found that serum glucose, insulin and HbA1c levels were 133.2 ± 7.2 mg/dL, 9.0 ± 0.2 µ/mL and 0.53 ± 0.02 mg/g Hb, respectively for date fruit extract-treated group as compared to 299.6 ± 16.7 mg/dL, 8.9 ± 0.7 µU/mL and 0.9 ± 0.05 mg/g Hb for untreated group (*p* < 0.05). Glucose metabolism was improved possibly due to the action of polyphenols such as flavonoids, anthocyanins and phenolic acids, which can detoxify free radicals and inhibit lipid peroxidation [57].

In an eight-week in vivo study by Al-Malki et al. [38], ethyl acetate extracts of *Balanites aegyptiaca* date were administered to diabetic rats. It was observed that the extracts significantly reduced glucose levels as compared to untreated diabetic rats (340.0 ± 15.8 vs. 280.0 ± 13.0; *p* < 0.05). It also significantly reduced HbA1c levels in treated diabetic rats compared to untreated diabetic rats (7.4 ± 0.8 vs. 6.6 ± 0.6; *p* < 0.05). In addition, vascular endothelial growth factor levels were significantly reduced in diabetic retina (*p* < 0.001), suggesting that date fruit may provide additional endothelial protection to the retina. In a separate study, alloxan-induced male Wister diabetic rats (*n* = 30) were experimented in a 10-day trial involving the administration of date fruits. A significant decrease in glucose level was observed in treated rats (*p* < 0.05) compared to untreated diabetic rats [58]. Two natural flavonoid compounds (diosmetin 7-O-β-L-arabinofuranosyl β-D-apiofuranoside and diosmetin 7-O-β-D-apiofuranoside) were isolated from date fruits and were administered in a group of male diabetic rats. Interestingly, Michael et al. [36] identified that the compounds were able to significantly (*p* < 0.01) reduce blood glucose levels from 330.0 ± 5.5 to 140.0 ± 1.2 and 158.0 ± 1.3 mg/dL, respectively indicating that both compounds have potentials in lowering blood glucose level.

#### 4.1.2. Antihyperglycemic Activity

In an in vivo study [58], it was observed that the effects of date palm fruit on lipid profile where significant, especially on cholesterol, low-density lipoprotein (LDL), and triacylglyceride (TG) in diabetic rats compared to that in untreated diabetic rats; however, no significant change in high-density lipoprotein (HDL) levels.

#### 4.1.3. Against Diabetes-Induced Testicular Toxicity

In an interesting eight-week study, Hosseinipour et al. [59] evaluated the alcoholic extract of Asrasi date on diabetes-induced testicular injuries on streptozotocin-induced male T2DM rats (*n* = 40). The levels of testis superoxide dismutase (SOD), glutathione peroxidase (GPx), catalase (CAT), and malondialdehyde (MDA) were ameliorated in diabetic rats in addition of recovering serum testosterone levels and *BCL-2* expression. Therefore, it is evident that Asrasi date extract with potential antioxidant effects can improve diabetes-induced oxidative stress and structural changes in the testis by strengthening the testicular antioxidant defence system.

#### 4.1.4. Against Diabetes-Induced Cardiomyopathy

In a remarkable in vivo study, Saddi et al. [46] observed that following the administration of *Phoenix dactylifera* extracts, the serum inflammatory molecules, tumour necrosis factor (TNF-α) and C-reactive protein were improved, besides scaling down the serum cardiac function enzyme: creatine phosphokinase-MB in diabetic rats compared to untreated diabetic rats. Additionally, increased levels of cardiac antioxidant enzymes including MDA, GPx were detected with attenuated the cardiac apoptosis enzyme: caspase-3 and the oxidative DNA fragmentation. Altogether, *Phoenix dactylifera* extracts were confirmed to possess pleiotropic protective mechanisms against diabetic cardiomyopathy including anti-diabetic, anti-inflammatory, antioxidant, and anti-apoptosis activities.

#### 4.1.5. Ameliorating Haematological Parameters

Zaakouk et al. [47] in his in vivo study concluded that date consumption can improve red blood cells (RBCs), white blood cells and haemoglobin (Hb) levels in diabetic rats compared to untreated diabetic rats, indicating that dates can restore diabetes-induced anaemic condition. Another in vivo study [49] also supports the fact that *Phoenix dactylifera* fruit extracts has the potential to significantly improve (*p* < 0.05) the RBCs and Hb level in diabetic rats.

#### 4.1.6. Neuroprotective Activity

In a six-week experiment on streptozotocin-induced diabetic rats, date fruit extract conferred significant improvement in diabetic neuropathy as compared to the control group [60]. In diabetic rats, oxidative stress causes reduction in vascular impairment as a result of endoneurial hypoxia, thus, contributing to impairment in neuronal function. Nevertheless, cinnamic acid, flavonoids, and vitamin C of date fruit extract can ameliorate the damages via their antioxidant and free radical scavenger activities. As a result, there is inhibition of the production of reactive oxygen species, which help to prevent oxidative stress and stimulate Schwann cells to produce nerve growth factors, helpful for neuronal support [61]. Similarly, Bendary et al. [56] also observed neuroprotective activities of date palm fruit, although the mechanism was unclear.

Taken together, the findings from the animal studies indicated that date consumption not only contributes to improvement in plasma glucose, HbA1c levels and insulin secretion, but also contributes to the protection of neurons, haematological biomarkers, cardiomyopathy, testicular toxicity, and improved pancreas along with retinal structure in animal models of T2DM.

### 4.2. Evidence from Clinical Studies

#### 4.2.1. Antihyperglycemic Activity

In the last 20 years, a few studies on date consumption in T2DM patients have been conducted (Table 2). In a randomised controlled trial [62], T2DM patients (*n* = 55) having blood glucose levels of more than 126 mg/dL were treated with date vinegar (20 mL) together with their normal diet for 7 weeks. Subsequently, it was observed that date vinegar significantly (*p* = 0.001) ameliorated the levels of HbA1c (6.8 ± 2.3 to 6.1 ± 2.1 (%)) and fasting blood sugar (171.4 ± 36.7 to 147.5 ± 38.8 mg/dL) indicating that date vinegar may be useful for diabetics. Acetic acid, which is the major component of date vinegar, may stall digestion of starch molecules in the small intestine by blocking disaccharide activity and reduce glucose uptake via muscle performance; it also responsible for gastric emptying. Additionally, fructose and dietary fibres may also be responsible for reducing blood glucose levels [63,64]. However, as this study was not on natural date fruit, the results should be used with caution in compared to those studies on natural date palm fruits. In order to identify the relationship between date fruit consumption and prevalence of developing T2DM, a large study on diabetic and non-diabetic Saudi patients (*n* = 2177) were conducted. This experiment revealed that there was no significant relationship between date consumption and the prevalence of developing T2DM. On the other hand, positive effects were observed since the dates provide dietary fibres and non-starch polysaccharides [65]. In another interesting study [66], hot water and sun drying treatments were utilised to reduce date sugar content. Both treatments could significantly reduce the sugar (fructose, glucose and inverted sugar) and mineral content (sodium, potassium, and calcium), making dates more suitable for diabetics. In a crossover clinical trial, Bam Mazafati dates and raisins were administered to patients with T2DM (*n* = 15) as a snack. After 2 h of snack (24.2 g or approximately 2 dates), it was noted that the dates did not significantly increase blood glucose levels (125.0 ± 18.9 (fasting), 161.2 ± 46.9 (2 h after breakfast) and 103.8 ± 20.9 mg/dL (2 h after date snack)) possibly due to the presence of the high polyphenol content [67]. Therefore, for diabetic patients, dates can be a nutrient-based beneficial snack as compared to sugar-based snacks [68].

Alkaabi et al. [69] evaluated the effects of traditional Arabic coffee consumption with Khalas date on glycaemic index in diabetic (*n* = 10) and healthy subjects (*n* = 13). Following a short-term (five days) experiment, there was no significant increase in the glycaemic index in the healthy individuals (52.7 ± 6.2 mg/dL) or in diabetic patients (41.5 ± 5.4 mg/dL). This phenomenon occurred possibly because of the presence of caffeine, which stimulates the secretion of epinephrine, which have an opposite action to insulin by acting via β-adrenergic stimulation [71]. Therefore, it was postulated that consumption of Arabic coffee along with Khalas dates can reduce blood glucose levels although a longer duration study is required to further confirm the findings. In another study [15], five varieties of dates (Fara’d, Lulu, Bo ma’an, Dabbas, and Khalas) were evaluated in diabetic and healthy individuals for eight days, by using 50 g of date flesh. There was no significant difference in the mean glycaemic indices of healthy and diabetic patients where the healthy individuals had 54.0 ± 6.1, 53.5 ± 8.6, 46.3 ± 7.1, 49.1 ± 3.6, 55.1 ± 7.7 while the diabetics had 46.1 ± 6.2, 43.8 ± 7.7, 51.8 ± 6.9, 50.2 ± 3.9, and 53.0 ± 6.0 for Fara’d, Lulu, Bo ma’an, Dabbas, and Khalas dates, respectively. The finding indicates that the date varieties do not increase the glycaemic indices in healthy or diabetic individuals.

The effect of dates which contained 25% fructose were evaluated in a clinical study with T2DM individuals (*n* = 16). Dates can decrease blood glucose levels when they were replaced with equal amount of bread in the breakfast (117.0 ± 21.6 mg/dL vs. 148.0 ± 32.4 mg/dL; *p* = 0.02 [70] possibly due to the rich presence of polyphenols and dietary fibres. Ahmed et al. [72] evaluated the glycaemic index in Saudi individuals (*n* = 19) who had Arabian breakfast like organic juice, boiled egg, hot milk, Arabic coffee, Arabic bread, and a date meal. It was noted that Khalas date meal was significantly better in terms of maintaining glycaemic index (57.7 mg/dL vs. 79.0 mg/dL) because it contains fructose as well as fibres [73]. Therefore, the study concluded that date meal is a beneficial diet for diabetic subjects as compared to conventional Saudi breakfast.

In a comparative clinical trial of type 1 DM patients (*n* = 20), a date (10 g) and a sugar cube (5 g) were administered to two groups of patients. Subsequently, their blood glucose levels were compared at 30, 60, 90, and 120 min. The mean blood glucose levels were not significantly different between the two groups (1619.4 ± 614.0 mg/dL (a date) and 1572.0 ± 967.0 mg/dL (a sugar cube)) after 2 h indicating that administration of dates to type 1 diabetic patients [74] is not recommended. Seventeen varieties of Saudi dates were administered to 19 patients with T2DM for evaluation of glycaemic index and load. Shaqra, Sukkary, and Sag’ai date varieties exhibited that the lowest glycaemic index ranging from 42.8 to 44.0 whereas, Ajwa and Shaqra conferred low glycaemic loads (from 8.5 to 9.2) [75]. These date varieties are rich in fructose and fibres, which may be responsible for reducing glycaemic index, glycaemic load, intestinal absorption, and gastric emptying that subsequently reduce the availability of α-amylase to its substrate, followed by a reduction in blood glucose level (Figure 1) [76]. Therefore, date varieties with lower glycaemic indices may be incorporated in the diet of diabetic individuals.

Taken together, even though date fruits are high in fructose, its consumption not only regulates plasma glucose concentrations, but also improves HbA1c and blood glucose levels. Therefore, it is plausible that daily sugary snacks can easily be substituted by date fruits, especially in patients with T2DM. However, due to the few clinical trials examining the effects of date fruits on T2DM patients, it is highly recommended for more trials to be conducted to determine the efficacy, safety as well as the amount of the recommended daily intake required for diabetic patients.

#### 4.2.2. Antihyperlipidemic Activity

In a clinical trial, Ali et al. [62] demonstrated that date vinegar can significantly (*p* < 0.05) improve cholesterol and LDL levels while significantly (*p* < 0.05) increasing HDL levels.

#### 4.2.3. Ameliorating Liver Function

Interestingly, a clinical trial confirmed that date palm can improve the liver functions in patients with T2DM by improving alkaline phosphatase (ALP) and alanine aminotransferase (ALT) levels without exerting any significant adverse effects [62].

## 5. Date Fruit Seed

### 5.1. Evidence from In Vivo Studies

#### 5.1.1. Antihyperglycemic Activity

Date seeds are promising source of nutrients, fibres and oil with functional properties [17,77,78]. In a 2-week in vivo study [79], date seed significantly reduced blood glucose levels and body weight (*p* < 0.005) of alloxan-induced diabetic rats when compared to control. Besides reduced glucose level, a significant (*p* < 0.05) increase in SOD, CAT, glutathione levels and a significant (*p* < 0.05) decrease in the MDA level were noted in the diabetic rats as compared to the untreated diabetic rats (444.3 ± 6.0 vs. 388.0 ± 4.5 mg/dL) administered with date palm seed extract [76]. Thouri et al. [80] detected in vivo glucose lowering and anti-inflammatory activities of Tunisian date seed which was eventually attributed to the presence of phenolics and flavonoids and antioxidant activities. The seed extracts of two Saudi date varieties (Ajwa and Sukkari) were evaluated in streptozotocin-induced diabetic rats in an eight-week experimental procedure. Following the intervention, blood glucose levels of diabetic rats which received Ajwa date seed extracts were significantly reduced (434.0 ± 20.0 mg/dL vs. 148.0 ± 28.7 mg/dL; *p* < 0.001) as compared to control. This is similarly seen in rats, which received Sukkari date seed extracts as compared to control (434.0 ± 20.0 mg/dL vs. 171.0 ± 9.2 mg/dL, *p* < 0.001). In addition, the two varieties also reduced diabetic rats’ body weight after eight weeks (Ajwa: 279.0 ± 2.1 g to 276.0 ± 7.4 g and Sukkari: 280.0 ± 1.0 g to 275.0 ± 3.1 g), thus, indicating that both varieties can reduce blood glucose levels and therefore have the potential to be investigated as anti-diabetics [81]. 

Abdelaziz et al. [82] estimated the effect of *Phoenix dactylifera* date seed in treating early complications of diabetes in streptozotocin-induced diabetic rats. Following the administration of aqueous suspension of date seed for four weeks, glucose level was decreased in date seed-treated rats when compared to untreated rats (248.0 ± 42.0 mg/dL vs. 508.0 ± 60.0 mg/dL). Khalil et al. [83] demonstrated that blood glucose level was significantly (*p* < 0.05) reduced from baseline (284.7 mg/dL vs. 172.5 mg/dL) following a 30-day administration with date seed powder (Zahdi variety) given in combination with fine bran to streptozotocin-induced diabetic rats. Furthermore, date seed supplementation at 5%,10%, and 15% can significantly reduce blood glucose levels in a concentration-dependent manner in male diabetic rats to 176.7 ± 11.0 mg/dL (*p* < 0.05), 130.7 ± 9.0 mg/dL (*p* < 0.01) and 121.1 ± 11.5 mg/dL (*p* < 0.001), respectively. It is plausible that the date seeds ameliorated glucose levels via its high dietary fibre and high chromium levels, essential for the synthesis of glucose tolerance factors. Date seed may also increase the activity of glucose-6-phosphate dehydrogenase by increasing insulin secretion, thus increasing the influx of glucose into pentose monophosphate shunt and reducing blood glucose levels [84]. In an eight-week in vivo experiment [85], oral administration of date seed extracts along with insulin demonstrated a significant (*p* < 0.05) antihyperglycemic effect on streptozotocin-induced diabetic rats as compared to administration of insulin alone. There was also a significant lowering (*p* < 0.05) of HbA1c levels due to stimulation of undifferentiated pancreatic islet cells to differentiate into newly formed β-cells. In fact, other research from a similar group demonstrated that date seed can stimulate endogenous insulin secretion from β-cell of pancreatic islets in type I diabetic rats [86]. Finally, date seed extract is reported to reduce blood glucose levels in male albino rats (*n* = 24) in a concentration-dependent manner. In the experiment, 10% and 15% date seed extracts which were mixed with fortified bread caused significant reduction in glucose levels from 152.5 ± 3.4 mg/dL to 119.8 ± 4.7 mg/dL (for the former) and to 105.6 ± 4.1 mg/dL (for the latter) [87]. It was hypothesized that the effects occur due to the high presence of the dietary fibres which are insoluble in water (including cellulose, hemicellulose and lignin), in addition to micro- and macro elements of date seed [88,89].

#### 5.1.2. Antihyperlipidemic Activity

Ayatollahi et al. [79] perceived that date seeds have the potential to significantly (*p* < 0.05) reduce LDL and cholesterol levels in diabetic rats as compared to the control group. Abiola et al. [76] and Khalil et al. [83] also detected a significant decrease (*p* < 0.05) in the levels of cholesterol, TG, and LDL with improved levels of HDL in diabetic treated rats as compared to the untreated diabetic rats. Similar results were depicted in other in vivo studies on diabetic rats [87,90]. Hasan et al. [81] detected Saudi date seeds have the potentials to reduce the levels of cholesterol and TG in diabetic rats compared to untreated diabetic rats.

#### 5.1.3. Against Diabetes-Induced Testicular Toxicity

Interestingly, in male rats administrated with date seed, testicular antioxidant enzyme status were dramatically improved [90] indicating that date seed has promising effects against diabetic-induced reproductive disorders. 

#### 5.1.4. Ameliorating Liver and Kidney Functions

In other multiple in vivo studies [79,87], date seeds exhibited the potential to reduce serum levels of creatinine, urea, and ALP in diabetic rats indicating date seeds can ameliorate kidney and liver functions in T2DM. In addition, no acute toxicity was detected even after high dosage of extract administration. Another study [82] also demonstrated that the levels of antioxidant enzymes including glutathione S-transferase, CAT and SOD were also significantly improved in both the kidneys and liver of date seed treated diabetic rats compared to the untreated rats. Subsequently, El Fouhil et al. [91] demonstrated that date seed extract was not only safe, but also minimised the toxic effects in the liver and the kidneys by improving ALT, aspartate aminotransferase, gamma glutamyl transferase, blood urea nitrogen, and creatinine levels.

Taken together, based on the T2DM animal model studies, date seeds are potential anti-diabetic agent due to its glucose and HbA1c lowering capacities in addition to improving the liver, the kidneys, reproductive system, and overall lipid profile.

### 5.2. Evidence from Clinical Studies

#### Antihyperglycemic Activity

Gharib et al. [92] evaluated the phenolic content and anti-diabetic effect of date kernels coffee among diabetic patients. Date kernels are a rich source of antioxidants due to the presence of numerous phenolic compounds including epicatechin, ellagic, chlorogenic, gallic, and caffeic acids. In this study, two cups of date kernels coffee in 200 mL were administered daily for three months (with each cup containing 10 g date kernels). After three months, fasting glucose to insulin ratio was significantly decreased (*p* < 0.001) with significant improvement in β-cell function (*p* ˂ 0.001). Therefore, due to the overall improvement of serum glycaemic profile, it was recommended that date kernel has the potentials to be incorporated into an anti-diabetic regimen. However, more clinical studies are required to fully establish this fact.

## 6. Date Fruit Leaf

### 6.1. Evidence from In Vivo Studies

#### 6.1.1. Antihyperglycemic Activity

In a recent study conducted on streptozotocin-induced diabetic male Wistar rats [93], when date leaf extract was administered orally, blood glucose, HbA1c and MDA levels were significantly decreased (*p* < 0.05), while plasma insulin along with a number of β-cells significantly increased (*p* < 0.05) in treated diabetic rats compared to control rats. Ismail et al. [94] observed after a 28-day follow-up that extracts of palm leave tops did not significantly decrease blood glucose levels in streptozotocin-induced diabetic Sprague–Dawley rats (before: 411.1 ± 84.2 mg/dL vs. after: 399.7 ± 172.2 mg/dL, *p* = 0.90) as compared to controls. The effect of phenolic compounds extracted from Iraqi date palm leaves were evaluated in alloxan-induced diabetic rabbits (*n* = 12) in a 24-h procedure [95]. When date palm leaf extracts were administered at different intervals, such as 2, 4, 6, and 24 h, blood glucose levels of the diabetic rabbits significantly decreased (392.3 ± 4.7 mg/100 mL to 325.5 ± 4.7 mg/100 mL (*p* < 0.05), 280.6 ± 2.7 mg/100 mL (*p* < 0.01), 238.3 ± 8.1 mg/100 mL (*p* < 0.01) and 134.5 ± 4.8 mg/100 mL (*p* < 0.001), respectively). The subsequent reduction indicates that date palm leaf extract has strong glucose-lowering effects in a time-dependent manner. Chakroun et al. [96] reported the presence of ten phenolic compounds in date palm leaf extract and conducted an in vivo study for 28 days in alloxan-induced diabetic mice. They identified that α-glucosidase and α-amylase enzymes were inhibited by the date palm leaves extract. Significant anti-diabetic activity was observed from date palm leaves extract when compared to Glucor^®^ (acarbose) administered at 50 mg/kg. This finding confirms that date palm leaf extract can reduce blood glucose levels and may have superior anti-diabetic effect as compared to Glucor^®^.

Following the administration of *Ziziphus jujube* (jujube date) leaf extract in alloxan-induced diabetic rats, there were significant (*p* < 0.001) reduction in blood glucose levels (from 767.8 mg/dL to 250.9 mg/dL) although when compared with glibenclamide-administrated group, the difference was not significant as reported by Eddine et al. [13]. The mechanism behind this discovery could be hypothesised as due to the contribution of jujube date in stimulating β-cells and activating insulin receptors and subsequently lowering the blood glucose level [97]. In an interesting study [98], upon subacute administration of *Phoenix dactylifera* leaf extract in alloxan-induced male Wister rats, blood glucose and insulin levels significantly improved. Similar results were confirmed by Abuelgassim et al. [99] where date palm leaf extract was incorporated in alloxan-induced male Wister diabetic rats. Subsequently after 4 weeks, there was a significant glucose reduction in diabetic rats (17.43 ± 0.76 to 16.77 ± 0.28 mmol/L/) as compared to controls. It is hypothesised that the leave extract promotes insulin secretion by closing the K^+^ ATP channels. Additionally, some components of leaves extract such as flavonoids, phenols, steroids and saponins have free radical scavenging abilities. It may also reduce water intake and improve body weight with a reverse dyslipidaemia effect [100,101].

#### 6.1.2. Antihyperlipidemic Activity

In an in vivo study, Mard et al. [98] demonstrated that upon administration of subacute *Phoenix dactylifera* leaf extract, both cholesterol and TG levels improved in diabetic male Wister rats compared to the control group. Abuelgassim et al. [99] also confirmed that administration of date palm leaf can significantly improve cholesterol (*p* < 0.001) and LDL levels (*p* < 0.001), however, no significant improvement on serum HDL levels.

#### 6.1.3. Ameliorating Haematological Parameters

Nuha et al. [95] reported from an in vivo study that the phenolic compounds identified in date leaves possessed no toxic effects on red blood cells.

Therefore, these results suggest that date leaves have potential roles in lowering plasma glucose, HbA1c, regulating lipid profile, protecting haematological parameters and, subsequently, improving diabetes and diabetes-associated complications in animal models.

## 7. Conclusions

Firstly, based on the in vivo and human studies, dates do not increase glucose levels, and glycaemic index in T2DM, rather, ameliorates diabetes-induced complications. Secondly, dates have the potential to lower glucose levels contributed by the polyphenols, flavonoids, and antioxidants. The possible mechanisms of actions were (i) stimulation of β-cell, (ii) increase in the number of islets and β-cells, (iii) decrease β-cell apoptosis and delay carbohydrate breakdown by inhibiting α-amylase, (iv) enhancement of α-glucosidase enzyme activities, and (v) reduce intestinal glucose absorption. Therefore, date glucose lowering activities should be explored further in larger studies and clinical trials for confirmation of its efficacy and safety in diabetic patients.

## Figures and Tables

**Table 1:** Polyphenols extracted from date fruits.

No.	Types of Date	Polyphenols	Country	References
1	Deglet Nour and Ftimi (*Phoenix dactylifera* L.)	Caffeoylshikimic acid hexoside 4-Caffeoylshikimic acid 5-Caffeoylshikimic acid 3-Caffeoylshikimic acid Caffeoylshikimic acid hexoside Caffeoyl-sinapoyl hexoside Dicaffeoyl-sinapoyl hexoside	Tunisia	[27]
2	Date palm (*Phoenix dactylifera* L.)	Ellagic acid Gallic acid p-Coumaric acid	Tunisia	[28]
3	Date palm pollen (*Phoenix dactylifera* L.)	Luteolin-7-O-β-D–glucoside Apigenin Isorhamnetin-3-O-glucoside Naringin	Egypt	[29]
4	Date palm (*Phoenix dactylifera* L.)	Gallic acid	Oman	[30]
5	Fardh, Khasab, and Khalas date fruits (*Phoenix dactylifera* L.)	Catechin	Oman	[31]
6	Date palm Ajwa, Barni (*Phoenix dactylifera* L.)	Hydroxybenzoic acid Hydroxycinnamic acid	United Kingdom	[32]
7	Date palm (*Phoenix dactylifera* L.)	Ferulic acid Sinapic acid	Morocco	[33]
8	Date palm (*Phoenix dactylifera* L.)	Caffeic acid Epicatechin Vanillic acid Coumarin Quercetin Rutin	Tunisia	[34]
9	Harvest date	Rutin Quercitrin Lisetin Myricetin Morin Luteolin Quercetin Apigenin, Kaempferol Isorhamnetin Rhamnetin Galangin	China	[35]
10	Date palm (*Phoenix dactylifera L.*)	Diosmetin 7-O-L-arabinofuranosyl-D-apiofuranoside Diosmetin 7-O-D-apiofuranoside	Egypt	[36]
11	Palm Date (*Phoenix dactylifera* L.)	Gallic acid Caffeic acid p-Coumaric acid Quercetin Ferulic acid Chlorogenic acid Sinapic acid Luteolin-7-O-rutinoside Apigenin-c-glycoside Quercetin3-O-rutinoside Protocatechuic acid p-Hydroxybenzoic acid Vanillic acid m-Coumaric acid o-Coumaric acid 5-o-Caffeoyl shikimic acid Cinnamic acid	Egypt	[37]
12	Desert date (*Balanites aegyptiaca*)	Vanillic acid Syringic acid β-sitosterol	Saudi Arabia	[38]
13	Desert date (*Balanites aegyptiaca*)	Epicatechin-O-glucoside Rutin Isorhamnetin-3-O-rutinoside Isorhamnetin-3-O-glucoside Quercetin Isorhamnetin	Egypt	[11]
14	Date palm (*Phoenix dactylifera* L.)	Salicylic acid kaempferol-3-glucoside p-Hydroxybenzoic acid Protocatechuic acid Vanillic acid Gallic acid Syringic acid o-Coumaric acid p-Coumaric acid Caffeic acid Ferulic acid Xanthoxylin acid Hydrocaffeic acid Coumaroylquinic acid Protocatechuic acid	Oman	[39]
15	Date Palm (*Phoenix dactylifera* L.)	Gallic acid p-Hydroxybenzoic acid Vanillic acid p-Coumaric acid Protocatechuic acid Syringic acid Caffeic acid Ferulic acid	Tunisia	[40]
16	Date syrup	Quercetin Epigallocatechin Gallate Curcumin Resveratrol	United Kingdom	[41]
17	Date palm (*Phoenix dactylifera L.*)	Isoquercitrin Luteolin 7-O-β-D-neohesperopyranoside 3j-O-methylether Luteolin 7-O-β-D-neohesperopyranoside Acacetin 7-O-β-D-neohesperopyranoside Apigenin 7-O-D-apiofuranoside Apigenin 7-O-D-apiofuranosyl-(1→2)-O-β-D-glucopyranoside Genistein 8-C-β-D glucopyranoside	Egypt	[42]
18	Barhee and Zahdi dates (*Phoenix dactylifera* L.)	Catechol 4 methyl catechol Chlorogenic acid Caffeic acid	Iraq	[43]
19	Deglet Nour (*Phoenix dactylifera* L.)	Procyanidin Protocatechuic acid Catechin Epicatechin Caffeoylshikimic acid Apigenin di-C-hexoside Hydroxycinnamic acid Quercetin Kaempferol hexoside	Australia	[44]
20	Date palm (*Phoenix dactylifera* L.)	Catechin Epicatechin Procyanidin B1 Procyanidin B2 Procyanidin A2	Australia	[45]

**Table 2:** Date consumption studies in human T2DM subjects.

Study ID [References]	Country	Study Design	Number of Participants (Female)	Date Consumption	Study Duration	Outcomes
Al-Mssallem 2018 [65]	Saudi Arabia	Cross-sectional	2177 (1133)	100 g/day	Weekly and monthly consumption of dates was recorded	Consumption of dates has no association with the prevalence of T2DM
Ali 2018 [62]	Pakistan	Double-blinded randomised-controlled trial	60 (29)	20 mL/day	10 weeks	Date vinegar improved blood concentrations of HbA1c and FBS (*p* < 0.05) in patients with T2DM
Foshati 2015 [68]	Iran	Non-randomised crossover clinical trial	15 (10)	24.2 g	3 days	Consumption of dates did not increase blood glucose
Alkaabi 2013 [69]	United Arab Emirates	Case-control	10 (5)	50 g	5 days	Dates exhibited as a low-GI fruit for patients with T2DM when consumed with and without Arabic coffee
Alkaabi 2011 [15]	United Arab Emirates	Non-randomised clinical trial	10	50 g	3 days	Diabetic individuals do not result in significant postprandial glucose excursions due to the date consumption
Forghani 2003 [70]	Iran	Non-randomised clinical trial	16	NR	2 days	Glucose level decreases substantially following replacing the bread content in a diabetic diet with dates

T2DM: type 2 diabetes mellitus; HbA1c: glycated haemoglobin; FBS: fasting blood sugar; GI: glycaemic index; NR: not reported.
